# Reliability of Alkaline Phosphatase for Differentiating Flare Phenomenon from Disease Progression with Bone Scintigraphy

**DOI:** 10.3390/cancers14010254

**Published:** 2022-01-05

**Authors:** Ji-hoon Jung, Chae-Moon Hong, Il Jo, Shin-Young Jeong, Sang-Woo Lee, Jaetae Lee, Byeong-Cheol Ahn

**Affiliations:** 1 Department of Radiology, College of Medicine, Hanyang University Guri Hospital, Guri 11923, Korea; hoon2510@nate.com; 2 Department of Nuclear Medicine, School of Medicine, Kyungpook National University, Daegu 41944, Korea; shahking@hanmail.net (C.-M.H.); eye12knowu@hanmail.net (I.J.); syjeong@knu.ac.kr (S.-Y.J.); swleenm@knu.ac.kr (S.-W.L.); jaetae@knu.ac.kr (J.L.)

**Keywords:** bone scintigraphy, flare phenomenon, alkaline phosphatase, bone metastasis, breast cancer, prostate cancer

## Abstract

**Simple Summary:**

Bone scintigraphy is the most widely used radionuclide technique to investigate bone metastasis, primarily due to its reasonable time and cost factor. However, it is important to recognize that bone scintigraphy to assess treatment response sometimes shows a “flare phenomenon”, which can be misinterpreted as disease progression. Distinction between flare phenomenon and disease progression could help in the decision to continue effective treatments in patients with flare phenomenon and to cease ineffective treatments and consider other salvage treatment plans in patients with disease progression. Despite many methods having been tried to answer this question, there was still no reliable way to differentiate between flare phenomenon and progression of bone metastases. Our results suggest that ALP is a useful serologic marker to differentiate flare phenomenon from disease progression on bone scintigraphy in breast or prostate cancer patients with bone metastasis.

**Abstract:**

The flare phenomenon (FP) on bone scintigraphy after the initiation of systemic treatment seriously complicates evaluations of therapeutic response in patients with bone metastases. The aim of this study was to evaluate whether serum alkaline phosphatase (ALP) can differentiate FP from disease progression on bone scintigraphy in these patients. Breast or prostate cancer patients with bone metastases who newly underwent systemic therapy were reviewed. Pretreatment baseline and follow-up data, including age, pathologic factors, type of systemic therapy, radiologic and bone scintigraphy findings, and ALP levels, were obtained. Univariate and multivariate analyses of these factors were performed to predict FP. An increased extent and/or new lesions were found in 160 patients on follow-up bone scintigraphy after therapy. Among the 160 patients, 80 (50%) had an improvement on subsequent bone scintigraphy (BS), while subsequent scintigraphy also showed an increased uptake in 80 (50%, progression). Multiple regression analysis revealed that stable or decreased ALP was an independent predictor for FP (*p* < 0.0001). ALP was an independent predictor for FP on subgroup analysis for breast and prostate cancer (*p* = 0.001 and *p* = 0.0223, respectively). Results of the study suggest that ALP is a useful serologic marker to differentiate FP from disease progression on bone scintigraphy in patients with bone metastasis. Clinical interpretation for scintigraphic aggravation can be further improved by the ALP data and it may prevent fruitless changes of therapeutic modality by misdiagnosis of disease progression in cases of FP.

## 1. Introduction

Metastatic cancer commonly affects the bones. In particular, bone metastasis is clinically important in breast and prostate cancers due to their high prevalence. More than 80% of metastatic bone diseases are derived from these two cancers [1]. A previous large population-based study reported that the five-year cumulative incidence of bone metastases was 30% in patients with advanced breast cancer [2]. At postmortem examination, more than 70% of patients who died of breast cancer had evidence of bone metastases [1]. In patients with advanced prostate cancer, bone metastasis occurred in approximately 65–70% [3]. At postmortem examination, the incidence of metastatic bone disease was 90% [4].

Patients with bone metastases frequently experience bone pain and skeletal-related events (SREs); that is, the development of pathologic fractures and spinal cord compression, and the need for palliative radiotherapy or orthopedic surgery [1]. Optimal treatment is aimed at delaying progression of bone metastases and reducing pain, preventing SREs, and improving quality of life. An assessment of the objective response of metastatic bone lesions to systemic therapies, such as endocrine and cytotoxic therapy, is difficult [5].

Bone scintigraphy (BS) is the most widely used radionuclide technique to investigate bone metastasis, primarily due to its reasonable time and cost factor. Additionally, BS provides for visualization of the whole skeleton whereas skeletal surveys can vary in the degree of inclusion of the appendicular skeleton. Although the role of BS has been reduced due to the development of PET, it is a commonly used imaging modality for monitoring therapeutic response in patients with bone metastasis. Bone lesions with a good response to treatment will demonstrate a reduced or vanished presence compared to the high uptake visualized on a previous BS [6].

However, it is important to recognize that BS to assess treatment response sometimes shows a “flare phenomenon” (FP), which can be misinterpreted as disease progression. FP is defined as an increase in the number and/or intensity of focal bone lesions after treatment in patient with bone metastases, and the metastatic lesions demonstrate improvement on later scintigraphy. Successful treatment reduces the metastatic tumor burden and induces repair processes in patients with metastatic bone lesions. In such situations, bone remodeling and formation occurs, resulting in an increased uptake on BS that can be visualized as FP [7]. Increased activity on BS may possibly indicate both FP and disease progression until the performance of subsequent BS, because a FP after successful treatment is indistinguishable from disease progression due to treatment failure [8]. Distinction between FP and disease progression could help in the decision to continue effective treatments in patients with FP and to cease ineffective treatments and consider other salvage treatment plans in patients with disease progression.

Previous studies attempted to distinguish FP from disease progression using bone turnover markers, such as cross-linked carboxy-terminal telopeptide of type I collagen (ICTP) and tartrate-resistant acid phosphatase isoform 5b (TRACP), because they were useful for accessing treatment response [9,10]. However, these markers have not yet been clinically applied or accepted. Among the various bone turnover markers, alkaline phosphatase (ALP) is the most widely used bone turnover marker. It has the advantage of being more convenient and less expensive to measure than other bone turnover markers [11]. The role of ALP has not been clearly understood, but it seems to induce a local concentration of inorganic phosphate and reduce the concentration of extracellular pyrophosphate. In addition, it is localized in the membrane of osteoblasts and, thus, represents the activity of osteoblast [12].

Therefore, this retrospective study was conducted to evaluate whether ALP can differentiate FP from disease progression.

## 2. Materials and Methods

### 2.1. Patients 

Between 2011 and 2018, data on patients who newly underwent systemic treatment for bone metastasis from breast or prostate cancer were collected from Kyungpook National University Hospital. Patients who underwent subsequent treatment modalities after the failure or resistance of previous systemic therapy, as well as those who underwent first-line treatment for the metastasis, were also included. 

Pretreatment and posttreatment (within six months, first follow-up) BS were performed routinely on each patient. Without treatment change, additional subsequent BS was undertaken to distinguish between disease progression and FP (second follow-up BS). FP was defined as an increased extent and/or number of hot spots as indicated by the first follow-up BS, followed by improvement on the second follow-up BS. Progression was defined as a greater uptake on the first follow-up than on the pretreatment BS, with the second follow-up BS also showing worsened findings [13]. According to these definitions, each patient was classified into flare or progression groups. BSs were interpreted by on-duty, board-certified nuclear medicine physicians (three interpreting physicians with a range of 10–20 years’ experience). The original reports were retrospectively reviewed.

In the case of breast cancer, 318 patients underwent a first follow-up BS. Patients in whom pretreatment (*n* = 20) or a second follow-up (*n* = 43) BS could not be obtained were excluded, as were patients with synchronous malignancy (*n* = 7) and those who underwent local radiotherapy (*n* = 9). Among these 239 patients, 101 showed increased bone lesion extent or a new lesion on the first follow-up BS, while others showed no change and decreased bone lesion extent (*n* = 102 and 36, respectively). 

In the case of prostate cancer, 315 patients underwent a first follow-up BS. Patients with unavailable data for pretreatment BS (*n* = 99), a second follow-up BS (*n* = 9), and ALP (*n* = 2) were excluded, as were patients with synchronous malignancy (*n* = 1) and those who underwent external radiotherapy to the bone metastasis (*n* = 7). Among these 197 patients, 59 showed increased bone lesion extent or a new lesion on the first follow-up BS, while the others showed no change and decreased bone lesion extent (*n* = 49 and 89, respectively). Finally, 160 patients with bone metastasis and increased bone lesion extent or a new lesion on a first follow-up BS were enrolled in the current study. 

The study protocol was approved by the ethics committee of the Kyungpook National University Hospital (KNUCH 2018-02-004). No informed consent was needed because of the retrospective design of the study.

### 2.2. Image Acquisition

During this follow-up period, computed tomography (CT) and BS were used to evaluate treatment response for metastatic bone lesions. BS was performed 3 to 5 h after intravenous injection of 740 MBq (20 mCi) technetium −99 m hydroxymethylene diphosphonate (HDP). Anterior and posterior planar whole-body images were acquired using a dual-headed gamma camera equipped with a low-energy high-resolution collimator (Infinia, GE, Milwaukee, WI, USA) and using 128 × 128 matrices and a 15% energy window around the 140 keV. Additional static images of areas of interest were obtained as needed. BS was reviewed retrospectively by an experienced nuclear medicine physician who was blinded to the clinical data.

Pre and posttreatment contrast-enhanced CT of the chest and abdomen were performed on all patients using a Siemens Somatom Sensation 10 scanner (Siemens, Forchheim, Germany) with a 3 mm slice thickness. For contrast enhancement, intravenous contrast medium (Ultravist 370; Shering AG, Berlin, Germany) was administered. The CT confirmed metastatic bone lesions that had been revealed on BS and also revealed extraosseous metastasis. All patients were divided typically into three subgroups classified by the radiologic pattern of bone metastasis: osteolytic, osteosclerotic, or mixed type [6].

### 2.3. ALP

Standard biochemistry parameters including serum ALP were routinely obtained from the cycle of systemic therapy. ALP was measured using an enzyme method (Hitachi Automatic Analyzer 7600; Hitachi, Tokyo, Japan). The following levels of ALP were obtained:

- Measurements were made at pre and posttreatment BS, showing an increased extent and/or new hot lesion. The interval between examination of BS and ALP was less than 10 days;
- The difference between baseline and follow-up ALP was marked as ∆ALP; ∆ALP = follow-up ALP (at first follow-up BS)—baseline ALP (at pretreatment BS);
- “∆ALP ratio” was defined as the ratio of ∆ALP to baseline ALP; ∆ALP ratio = “∆ALP”/“baseline ALP” × 100 (%);
- Given the Hitachi Automatic Analyzer has 90–110% accuracy, “increased ALP” was defined as an increase in ∆ALP ratio of more than 10% of baseline;
- “Decreased ALP” was defined as a decrease in ∆ALP ratio of more than 10% of baseline;
- “Stable ALP” was defined as the remainder, being neither increased ALP nor decreased ALP.

### 2.4. Statistical Analysis

Continuous variables were expressed as the median and range, while categorical variables were expressed as numbers and percentages. For the univariate analysis, the median values of the two groups were compared statistically using a Mann–Whitney U test, while binomial variables were compared using a chi-squared test. Receiver operating characteristic (ROC) curve analysis was used to quantify the predictive ability of each cutoff value. Logistic regression analysis was used in the multivariate analysis to predict FP versus disease progression. In logistic regression, the odds ratio (OR) with 95% confidence intervals (CI) quantifies the strength of the association between two events and represents the constant effect of a predictor, or the likelihood that one outcome will occur. Subgroup analysis was performed for each group, which were classified according to the type of primary cancer. Statistical analyses were performed using Medcalc version 19.6.1 (Medcalc Software, Ostend, Belgium). All *p* values reported were two-sided, with values of <0.05 considered statistically significant.

## 3. Results

### 3.1. Patient Characteristics 

The characteristics of the 160 patients of the current study sample are summarized in Table 1. The median age of all patients was 58 years. Patients with prostate cancer were significantly older than those with breast cancer (median ages of 69 vs. 54 years). Bone metastases were diagnosed at initial presentation in 30 patients and at disease recurrence in 130. 

All breast cancer patients with recurrent bone metastasis underwent neoadjuvant or adjuvant chemotherapy and 42 underwent systemic therapy for distant metastasis. Among the 42 patients, hormone therapy (24.8%) was the most common prior therapy, followed by chemotherapy (20.8%), bisphosphonates (BPs, 11.9%), and HER2-targeted therapy (7.9%). In the current study, breast cancer patients underwent various therapies with a single regimen, including chemotherapy (*n* = 29), hormone therapy (*n* = 11), and HER2-targeted therapy (*n* = 2), or with a combination regimen, including hormone plus BPs therapy (*n* = 26), chemotherapy plus BPs therapy (*n* = 13), chemotherapy plus HER2-targeted therapy (*n* = 10), and chemotherapy plus HER2-targeted and BPs therapy (*n* = 9).

All 47 prostate cancer patients with recurrent bone metastasis underwent androgen deprivation therapy (ADT) except for three who received docetaxel following ADT. For prostate cancer, single chemotherapy (*n* = 29), chemotherapy with BPs therapy (*n* = 4), single ADT (*n* = 10), combined ADT (*n* = 10), combined ADT with BPs therapy (*n* = 1), combined chemotherapy and ADT (*n* = 1), and combined chemotherapy and ADT with BPs therapy (*n* = 4) were performed.

At bone metastasis diagnosis, 59 patients (36.6%) had coexistent extraosseous metastasis. In breast cancer patients, additional metastasis was found most frequently in the liver (*n* = 26), followed by lung (*n* = 23), pleura (*n* = 6), brain (*n* = 3), skin (*n* = 2), adrenal gland (*n* = 1), extraocular muscle (*n* = 1), and peritoneum (*n* = 1). In prostate cancer patients, additional metastasis was found most frequently in the lung (*n* = 4), followed by liver (*n* = 4) and pleura (*n* = 1). Extraosseous metastasis was more frequent in breast cancer patients (50.5%) than in those with prostate cancer (13.6%). 

**Figure 1:**
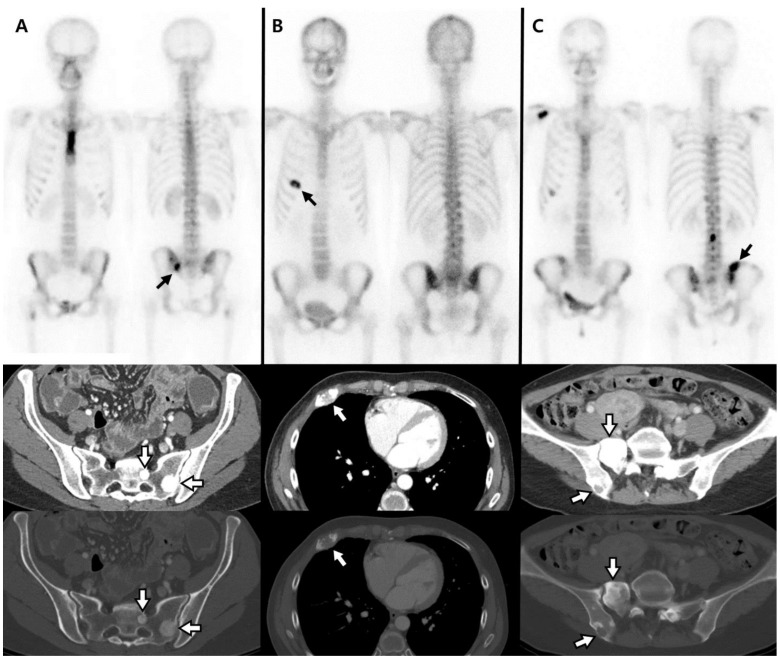
Examples of three subgroups classified by radiologic pattern. (**A**) Metastatic bone lesion in the left ala of the sacrum, which had increased radiotracer uptake on bone scintigraphy (black arrows), showed osteosclerotic change on CT image (white arrows). (**B**) Bone metastasis in the anterior arc of the right fifth rib showed increased radiotracer uptake with central photon deficiency on bone scintigraphy (black arrow) and osteolytic metastasis on CT image (white arrow). (**C**) Hot lesions were seen in the right iliac bone and sacrum on bone scintigraphy (black arrow), while on CT image (white arrows), metastatic lesion in the right iliac bone showed osteolytic change and, in the sacrum, osteosclerotic change (mixed type of bone metastasis).

Median times from pretreatment BS to the initiation of treatment and from the initiation of treatment to posttreatment BS were 1 and 2 months, respectively. Before initiation of treatment, 113 patients (62.4%) had osteosclerotic, 15 (10.9%) osteolytic, and 32 (26.7%) mixed bone metastases (Figure 1). Although osteosclerotic metastases were predominant in both types of cancer, prostate cancer showed a higher proportion of osteosclerotic change compared to breast cancer. The number of patients classified as flare group and progression group were both 80. 

Pathologic data for each patient were also obtained. Only three breast cancer patients (two with invasive lobular and one with invasive micropapillary carcinoma) did not have an invasive ductal carcinoma. Of the breast cancer patients, 76 (75.2%) had estrogen receptor, 57 (56.4%) had progesterone receptor, and 29 (28.7) had HER2 (28.7%). The number of prostate cancer patients with Gleason scores of 6 to 10 were 4 (6.8%), 5 (8.5%), 17 (28.8%), 22 (37.3%), and 7 (11.9%), respectively. Four patients did not have available Gleason score data. 

### 3.2. Univariate Analyses for Distinguishing FP from Disease Progression

Table 2 shows the results of univariate analysis of clinical, pathologic, scintigraphic, and biologic markers to distinguish between flare and progression groups. Clinical factors, including age, coexistence of extraosseous metastasis, history of systemic treatment, and administration of combination therapy, were not different between the two groups (*p* = 0.2836, *p* = 0.2529, *p* = 0.8379, and *p* = 0.8736, respectively). There were no significant differences in treatment regimens for metastatic bone disease between the two groups in regard to chemotherapy (*p* = 0.7463), hormone therapy (*p* = 0.3344), and BPs (*p* = 0.4106).

Overall, the pattern of scintigraphic change was similar between the two groups. Most patients in both groups had an increased extent of hot lesions (flare, 67 [83.8%]; progression, 64 [80.0%]; *p* = 0.5394). The new appearance of hot lesions was noted in 44 (55.0%) and 41 (51.3%) patients in flare and progression groups, respectively (*p* = 0.6357). Multiple scintigraphic changes were seen in 50 (62.5%) and 46 (57.5%) patients, respectively (*p* = 0.5199). The distributions of aggravated lesion on BS were not different between the two groups. In both groups, 16 patients had aggravated lesions in appendicular in addition to axial bones. Aggravated lesions in the pelvis were more prevalent in the flare group than the progression group (65.0% vs. 50.0%), but the difference was not statistically significant (*p* = 0.0557). Neither radiologic pattern was different between the two groups. On pretreatment CT image, osteosclerotic change was seen in 55 (68.8%) and 58 (72.5%) patients in flare and progression groups, respectively (*p* = 0.7239).

The median pre and posttreatment ALP levels in the flare group were 100 (range, 30–762) and 90 (42–619) IU/L, respectively, while those in the progression group were 81 (28–239) and 88 (36–475) IU/L, respectively. Although higher baseline ALP was noted in the flare group, it was not a factor for predicting FP due to the wide range of ALP in both groups. In analyses of ALP change by treatment, ∆ALP and the ratios of ∆ALP were significantly different between the two groups (*p* < 0.0001 and *p* < 0.0001).

**Figure 2:**
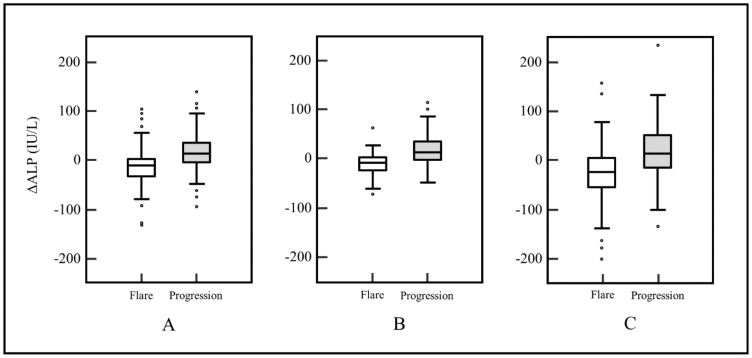
Comparison graphs for ∆ALP between flare and progression groups. ∆ALP was significantly lower in the flare group than in the progression group. Median ∆ALP was (**A**) −11 and 15 IU/L (*p* < 0.0001), respectively, in all patients; (**B**) −7 and 15 IU/L (*p* < 0.0001), respectively, in breast cancer patients; and (**C**) −23 and 15 IU/L (*p* = 0.0083), respectively, in prostate cancer patients.

**Figure 3:**
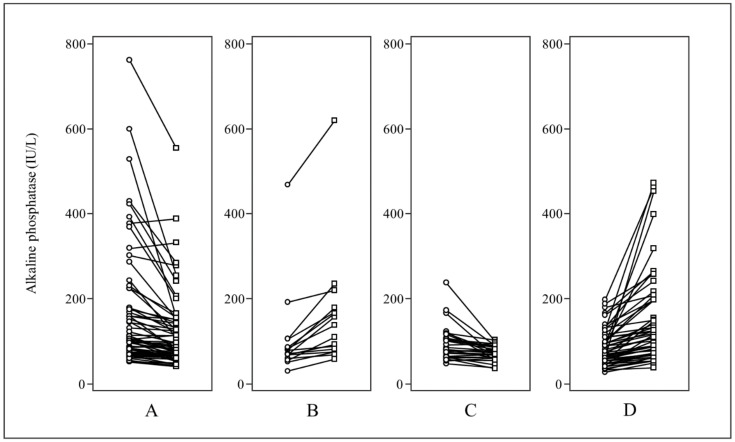
Dot and line graphs for directional changes in ALP. The dot and line graphs show individual directional change in the ALP of patients in the flare group for (**A**) stable/decreased ALP and (**B**) increased ALP, and in the progression group for (**C**) stable/decreased ALP and (**D**) increased ALP. The incidence of stable/decreased ALP was higher in the flare group than in the progression group (64/80, 80.0% vs. 29/80, 36.3%).

In subgroup analysis for breast and prostate cancers, ∆ALP was significantly different between the two groups (*p* < 0.0001 and *p* = 0.0083, respectively; Figure 2). The incidence of stable or decreased ALP level was significantly different between flare and progression groups (80.0% and 36.3%, respectively; *p* < 0.0001). FP occurred in 64 of 93 patients without an increased ALP (68.8 %), compared to only 16 of 67 (23.9 %) with an increased ALP. In addition, 41 of 80 (51%) patients in the flare group had decreased ALP levels over 10% compared to 17 of 80 (21%) in the progression group. Figure 3 shows the directional changes in ALP levels.

### 3.3. Multivariate Logistic Regression Analysis for Occurrence of FP

Table 3 shows the multivariate logistic regression analysis for occurrence of FP. Clinical factors, including age, presence of extraosseous metastasis, history of systemic therapy, and administration of combination therapy, were not significantly related with flare (*p* = 0.4105, *p* = 0.1333, *p* = 0.7670, and *p* = 0.6871, respectively).

There was no significant relationship between the incidence of flare and variety of systemic treatment: chemotherapy (*p* = 0.5965), hormone therapy (*p* = 0.6057), and BPs (*p* = 0.7632). Scintigraphic change, such as aggravating pattern and the number and location of aggravated lesions, was not associated with the occurrence of flare. Radiologic pattern was also not related to FP.

Change in ALP levels was an independent predictor of FP. Patients without increasing ALP (i.e., stable or decreased ALP) had a significantly higher odds of flare than patients with increasing ALP (OR = 10.6305; 95% CI, 4.4352–25.4800; *p* < 0.0001). In other words, patients with increasing ALP had 10-fold odds of progression than those without an increased ALP.

**Figure 4:**
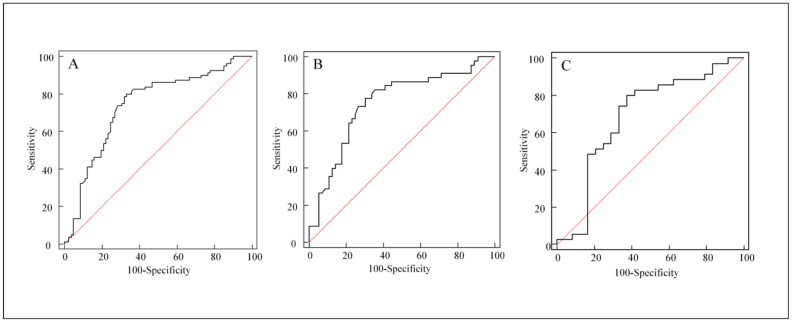
ROC curves for predictability of ∆ALP ratio to distinct flare phenomenon from progressive disease. The cutoff value was a ∆ALP ratio of 10% related to a definition of “increasing ALP,” not a Youden index. (**A**) In all patients, the ROC curve shows 0.735 of the area under curve (AUC), 80.0% sensitivity, and 63.8% specificity (*p* < 0.0001). (**B**) In breast cancer patients, the ROC curve shows 78.8% sensitivity and 66.1% specificity (AUC = 0.750, *p* < 0.0001). (**C**) In prostate cancer patients, the ROC curve shows 82.9% sensitivity and 58.3% specificity (AUC = 0.686, *p* = 0.0148).

In subgroup analysis for breast and prostate cancers, stable or decreased ALP level was still an independent predictor of FP (OR = 9.0827, *p* = 0.001, and OR = 18.5185, *p* = 0.0223, respectively). In prostate cancer, increasing prostate-specific antigen (PSA) levels did not demonstrate predictive value for progressive disease (OR = 0.4215; 95% CI, 0.0356–4.9891; *p* = 0.4932). Increasing ALP was the only factor that could predict progressive disease. The ROC curve demonstrates the change in ALP predicting FP in all patients, and in subgroups of breast and prostate cancer patients (Figure 4).

## 4. Discussion

Although there was still no reliable way to differentiate between FP and the progression of bone metastases, many methods have been tried to answer this question. Coleman et al. suggested that symptomatic deterioration might indicate disease progression [14]. Responders classified by the Union for International Cancer Control (UICC) criteria showed symptomatic benefit with a reduction in symptom score of >10%, while an increased symptom score was demonstrated in patients classified as having progressive disease.

However, a rapid increase in pain score after treatment of bone metastasis, called “clinical flare” or “pain flare,” was reported in previous studies [15,16]. After antiestrogen therapy for breast cancer, clinical flare, which is characterized by erythema in soft-tissue lesions and increased pain in bone and soft-tissue lesions, was reported. This clinical flare was reportedly due to edema of the periosteum of the metastatic bone lesion, resulting in nerve compression or the release of inflammatory cytokines [17]. It is certain that the worsening of symptoms cannot indicate progression in this situation. In addition, the measurement of symptoms is so subjective that standardization and quantification are difficult [14].

The development of a sclerotic rim or recalcification in an osteolytic lesion indicates a healing response to therapy, whereas the enlargement of an existing osteolytic lesion usually indicates disease progression [8]. Vassiliou et al. reported that osteosclerotic change, quantified by calculating the change in Hounsfield units within metastatic lesions, could provide a valid objective measure of treatment response following radiotherapy and BPs therapy [18]. The UICC and World Health Organization classification systems also include the appearance of calcifications in osteolytic bone lesion on CT as response [6]. Some studies supposed that increased osteoblast activity in osteosclerotic lesions or recalcification following successful treatment can result in increased accumulation of bone-seeking agents. Based on this hypothesis, the interval changes in the appearance of metastatic bone lesions on radiologic image, mainly CT, may validate the diagnosis of FP [5].

However, these studies had critical selection bias in that they evaluated only patients with osteolytic metastasis. FP occurs not only in osteolytic lesions, but also osteosclerotic lesions. Indeed, FP often is observed on BS in patients with bone metastasis from prostate cancer, which predominantly develops osteosclerotic feature [19,20]. In the current study, 49% of 113 patients with osteosclerotic metastasis had FP. Therefore, CT imaging cannot resolve the dilemma of differentiation between flare and disease progression in all bone metastasis patients. Additionally, conventional CT is limited in assessing the whole skeleton, especially upper and lower extremities. In the current study, 20% of patients had bone metastasis on extremities.

There are bone turnover markers which are classified into bone resorption markers and bone formation markers for assessing response [21,22,23,24]. Urinary calcium and hydroxyproline excretion are traditional bone resorption markers; however, urine concentrations poorly correlate with standard measures of systemic therapeutic response. ICTP, which are released from type I collagen by various proteolytic enzymes, during bone resorption and dissolution of the organic bone matrix, are associated with a response to anti-tumor therapy. Koizumi et al. reported that a marked increase in ICTP after combined chemotherapy of cyclophosphamide, doxorubicin, and 5-fluorouracil indicated disease progression, while a minimal change of ICTP indicated FP [10]. TRACP directly reflects osteoclast activity because it is a lysosomal enzyme secreted specifically by osteoclasts. Tsai et al. suggested that decreased serum TRACP, which could reflect osteoclast activity, may indicate FP [9]. They investigated direct interval change in TRACP and tracer uptake in BS using a semiquantitative bone scintigraphy index (SQBSI). Changes in TRACP and SQBSI had statistical significance in association with treatment response of bone metastasis in breast cancer. In some patients, while gradually declining TRACP was observed, the SQBSI actually increased. These discordant directional changes in TRACP and SQBSI indicated FP [9]. Bone formation markers such as ALP, osteocalcin, and amino-terminal and carboxy-terminal byproducts are increased in patients with bone metastases. These markers are associated with serial phases of proliferation with synthesis of type I collagen, maturation of the bone matrix, and mineralization [25,26]. In the current study, due to the retrospective study design, evaluation was possible only for serum ALP, which was routinely obtained. It could not be evaluated whether other markers could compensate for the low AUC value of our study. To overcome this limitation, further prospective studies of comparative and combinatorial analysis using other markers are warranted.

It is worthwhile noting that this is the first study to report that bone formation marker could be useful to differentiate FP from disease progression, as well as analyzing the most widely used bone turnover marker. Activated osteoblasts usually develop bone formation, thus, ALP values are positively correlated with BS findings. However, aggravation on BS may occur in good and poor responders because BS can reflect the bone formation and not the osteoclast or osteoblast activity. Using autoradiography, the mineralization front of bone (osteoid) and osteocytic lacunae were the deposition sites of methylene diphosphonate, but not near osteoclast or osteoblast [27].

In good responders, decreased osteoclast activity can develop into predominant osteoblastic activity, which leads to bone formation, without needing to increase osteoblast activity [5]. In the progressive metastatic bone lesion, osteoclasts and osteoblasts tend to increase, resulting in new destructive bone lesions [14]. Consequently, increased ALP was observed in patients with progression, while no significant change or even decreased ALP was observed in patients who responded to effective treatment.

Increased ALP levels demonstrating aggressiveness were reported in not only patients with bone metastasis, but also patients without bone metastasis. Tumor-derived ALP could also cause an increase in ALP during disease progression due to treatment failure. Aminian et al. found that an elevated ALP prominently occurred in esophageal cancer patients with lymph node involvement, and then they suggested that ALP levels can be associated with tumor proliferation [28]. In breast cancer, serum ALP activity was significantly decreased after surgery [29]. These results suggested that the primary cancer cell itself may increase ALP.

In the current study, ALP levels were increased in only 20% of patients with flare, compared to 64% of patients with progression (Figure 3). This result could suggest that patients with an increase in hot lesions and ALP levels may actually have progression rather than FP. Distinction between disease progression and FP could help in the decision to cease ineffective treatments and consider other salvage treatment plans for patients with disease progression and to continue effective treatments for patients with FP.

In the current study, “change of ALP” was used as the predictor of FP instead of an absolute ALP value for the following reasons. First, levels of serum ALP were too broad to define an ‘abnormal’ increase of serum ALP. The upper limit of a population-based reference range was relatively insensitive as the diagnostic cut-off limit. In patients with bone metastasis of breast cancer, 80% of patients demonstrated serum ALP levels in the reference range [30]. In the current study, serum ALP was reported from 28 to 762, though the majority of patients showed serum ALP within the reference range. Second, the current study showed a significantly higher level of baseline and follow-up ALP in patients with prostate cancer than those with breast cancer. This might be related to a higher incidence of osteosclerotic change in bone metastases of prostate cancer than those of breast cancer. Among the patients with the same type of primary cancer, it was reported that bone formation markers were dramatically higher in patients with osteosclerotic metastases [31,31]. Thus, these pathologic and biochemical differences among metastatic bone lesions emphasized that the change in serum ALP in each patient was informative for differentiating FP from disease progression, while the absolute value of serum ALP was not.

Patients with FP who respond well to hormone therapy may experience unnecessary side effects when changing the treatment strategy and receiving chemotherapy. Additionally, they may forfeit the chance of receiving chemotherapy in the future. In the current study, 130 (81.3%) patients had a history of systemic treatment before the current study. These patients who have experienced multiple treatment failures may have no or few applicable therapeutic options [32]. In breast cancer, when the disease is resistant to combination therapy containing luteinizing hormone-releasing hormone analogue and tamoxifen, alternative regimens for endocrine therapy are currently not available; when the disease is resistant to taxane-based chemotherapy, there are few therapeutic options [33,34]. In prostate cancer, docetaxel was proved to be effective against CRPC but there are limited regimens for docetaxel-resistant prostate cancer [35]. Physicians should carefully decide the discontinuation of therapy by taking into account the possibility of flare. The result of the current study may reduce erroneous changes of treatment modality by FP of BS.

The current study has several limitations. First, total ALP was used and not bone-specific ALP (bALP), which more directly reflects osteoblast activity. ALPs are ubiquitously present in the liver, intestine, kidney, placenta, and white blood cells, as well as bone. In the serum, bone and liver isoforms predominate in approximately equal amounts [36]. Because bALP is specifically present on the surface of osteoblasts, a previous study suggested that bALP seemed to be a better marker of bone mineralization than total ALP [37]. However, total ALP still is considered a significant bone turnover marker and is most widely used to assess treatment response in patients with bone metastasis [11]. Garnero et al. reported the high correlation between bALP and total ALP, with a correlation coefficient of 0.98 [38]. Total ALP was as sensitive as bALP; thus, this nonspecific bone turnover marker can be a valuable index of osteoblast activity.

Second, patients underwent various and complex regimens of systemic treatment in the current study. These various treatments affected and controlled various cells or pathways. It was impossible to determine the specific individual effects of each treatment. Thus, nonuniform treatment modalities could disturb the interpretation of FP. For example, it was considered that patients may have disease progression after treatment but relatively stable or even improved bone status due to concurrent BPs therapy [9]. To adjust for treatment-related variables, multivariate analyses were performed including these variables. The current study reported that the change in ALP was an independent predictor of FP even considering the treatment regimen.

Third, only qualitative analysis of BS was performed. The bone scan index (BSI) is a computer-assisted quantitative analysis of BS, which represents the total tumor burden as the fraction of total skeleton weight. After each metastatic hot spot is determined, the BSI is calculated as the sum of all such fractions using software (EXINI bone in Europe and North America; BONENAVI in Japan) [39]. The BSI has shown clinical impact as a prognostic biomarker in patients with prostate cancer [40,41]. It was reported that the BSI more appropriately reflected treatment response than visual interpretation [42]. There was a relationship between the changes in BSI and in several bone metabolic markers [43]. In the current study, the location and pattern of aggravated lesions were analyzed, which was not possible with BSI. Because the BSI is the sum of all fractions, it is difficult to conduct lesion-based analysis. According to the definition of FP and progression, it seems that a more reliable classification will be possible if quantitative analysis is performed in addition to the existing qualitative analysis.

Fourth, this study was limited by the small number of patients from a single center. Despite reviewed data for approximately 10 years, few patients were suited to the inclusion criteria. Furthermore, due to the design of the current study, all inherent biases associated with the retrospective design were possible. A large-scale and multicenter study with prospective design will be necessary to enhance the reliability and objectivity of current results.

To the best of our knowledge, this is the first study to suggest that a change in ALP level after systemic treatment was independently relevant to FP in patients with bone metastasis. The current study suggests that FP should be considered first, when BS shows aggravation but ALP is stable or decreased.

## 5. Conclusions

In the current study, 45 (19%) of 239 breast cancer patients and 35 (18%) of 197 prostate cancer patients, totaling 80 (18%) of all 436 patients, showed FP. It was suggested that serum ALP might be a useful serologic marker to differentiate FP from disease progression on BS in breast or prostate cancer patients with bone metastasis. Although the statistical evidence was rather weak in patients with prostate cancer, stable or decreased ALP was an independent predictor for FP. Interpretation of BS can be improved by considering the change in ALP, as this may reduce the occurrence of unnecessary changes in treatment modality. Results of the current study suggest that assessing response to systemic therapy for bone metastasis should consider the ALP change during therapy. Large-scale and multicenter studies with a prospective approach are required to confirm that changes of ALP will allow FP to be distinguished from disease progression.

## Figures and Tables

**Table 1:** Patient characteristics.

Characteristic	Total N = 160	Breast N = 101	Prostate N = 59	*p* Value
Age (years, range)	58 (32–84)	54 (32–82)	69 (47–84)	*p* < 0.0001
Clinical factor (%)				
At diagnosis of primary tumor	30 (18.6)	18 (17.8)	12 (20.3)	*p* = 0.7322
Extraosseous metastasis	59 (36.6)	51 (50.5)	8 (13.6)	*p* < 0.0001
On study therapy (%)				
Chemotherapy	98 (61.3)	61 (60.4)	37 (62.7)	*p* = 0.7724
Endocrine therapy	64 (40.0)	37 (36.6)	27 (45.8)	*p* = 0.2569
Bisphosphonate	57 (35.6)	48 (47.5)	9 (15.3)	*p* < 0.0001
HER2-targeted therapy		22 (21.8)	–	
Time interval (months, range)				
From pretreatment BS to treatment	1 (0–4)	1 (0–4)	1 (0–4)	*p* = 0.7798
From treatment to posttreatment BS	2 (0–6)	2 (0–6)	2 (0–6)	*p* = 0.3793
Radiological pattern (%)				
Osteosclerotic	113 (70.6)	63 (62.4)	50 (84.7)	*p* = 0.0086
Osteolytic	15 (9.4)	11 (10.9)	4 (6.8)	
Mixed	32 (20.2)	27 (26.7)	5 (8.5)	
Response to treatment (%)				
Flare phenomenon	80 (50.0)	45 (44.6)	35 (59.3)	*p* = 0.0724
Progression	80 (50.0)	56 (55.4)	24 (40.7)	
ALP (IU/L, range)				
Baseline ALP	89.5 (28–762)	75 (28–600)	113 (61–762)	*p* < 0.0001
Follow-up ALP	89.5 (36–619)	77 (36–475)	114 (56–619)	*p* < 0.0001
Receptor (%)				
Estrogen receptor-positive		76 (75.2)	–	
Progesterone receptor-positive		57 (56.4)	–	
HER2-positive		29 (28.7)	–	
Gleason score (%)				
6		–	4 (6.8)	
7		–	5 (8.5)	
8		–	17 (28.8)	
9		–	22 (37.3)	
10		–	7 (11.9)	
Unknown		–	4 (6.8)	

HER2, Human epidermal growth factor receptor 2; BS, bone scintigraphy; ALP, alkaline phosphatase.

**Table 2:** Univariate analysis between flare and progression groups.

Variables	Flare	Progression	*p* Value
	*N* = 80	*N* = 80	
Clinical factors			
Age (years, range)	60 (32–81)	56 (33–84)	*p* = 0.2836
Extraosseous metastasis (%)	33 (41.3)	26 (32.5)	*p* = 0.2529
Prior therapy (%)	66 (82.5)	65 (81.3)	*p* = 0.8379
Combination therapy (%)	45 (56.3)	46 (57.5)	*p* = 0.8736
Treatment regimen (%)			
Chemotherapy	50 (62.5)	48 (60.0)	*p* = 0.7463
Endocrine therapy	29 (36.3)	35 (43.8)	*p* = 0.3344
Bisphosphonate	26 (32.5)	31 (38.8)	*p* = 0.4106
Patterns of scintigraphic change (%)			
Extent	67 (83.8)	64 (80.0)	*p* = 0.5394
New lesion	44 (55.0)	41 (51.3)	*p* = 0.6357
Number of aggravated lesions (%)			
Multiple change	50 (62.5)	46 (57.5)	*p* = 0.5199
Location of aggravated lesions (%)			
Skull	9 (11.3)	15 (18.8)	*p* = 0.1854
Spines	45 (56.3)	48 (60.0)	*p* = 0.6318
Chest cage	49 (61.3)	41 (51.3)	*p* = 0.2038
Pelvis	52 (65.0)	40 (50.0)	*p* = 0.0557
Extremities	16 (20.0)	16 (20.0)	*p* = 1.0000
Radiologic patterns (%)			
Osteosclerotic	55 (68.8)	58 (72.5)	*p* = 0.7239
Osteolytic	7 (8.8)	8 (10.0)	
Mixed	18 (22.5)	14 (17.5)	
ALP			
Baseline ALP (IU/L, range)	100 (30 –762)	81 (28–239)	*p* = 0.0013
Follow-up ALP (IU/L, range)	90 (42–619)	88 (36–475)	*p* = 0.6159
∆ALP (IU/L, range)	−11 (−373–150)	15 (−140–393)	*p* < 0.0001
∆ALP ratio (range)	−10% (−71–132%)	19% (−60–644%)	*p* < 0.0001
Stable or decreased ALP level (%)	64 (80.0)	29 (36.3)	*p* < 0.0001

ALP, alkaline phosphatase.

**Table 3:** Multivariate logistic regression analysis for occurrence of the flare phenomenon.

Variable	Estimate	Standard Error	Wald χ^2^	*p* Value	OR	95% CI
Clinical factor						
Age > 60	0.32492	0.43791	0.5505	0.4581	1.3839	0.5866–3.2649
Extraosseous metastasis	0.60738	0.42946	2.0002	0.1573	1.8356	0.7911–4.2593
Prior therapy	0.17686	0.49504	0.1276	0.7209	1.1935	0.4523–3.1492
Combination therapy	−0.26917	0.55802	0.2327	0.6295	0.7640	0.2559–2.2808
Treatment regimen						
Chemotherapy	−0.78687	1.01881	0.5965	0.4399	0.4553	0.0618–3.3535
Hormone therapy	−0.77529	1.08234	0.5131	0.4738	0.4606	0.0552–3.8424
Bisphosphonate	−0.15488	0.53587	0.08354	0.7726	0.8565	0.2996–2.4484
Bone scan pattern						
Extent	0.52625	0.62111	0.7179	0.3968	1.6926	0.5010–5.7180
New lesion	0.49799	0.51028	0.9524	0.3291	1.6454	0.6052–4.4733
Number of aggravated lesions						
Multiple change	−0.55776	0.61424	0.8246	0.3639	0.5725	0.1718–1.9082
Location of aggravated lesions						
Chest cage	0.88091	0.46871	3.5323	0.0602	2.4131	0.9629–6.0471
Spines	0.28031	0.47020	0.3554	0.5511	1.3235	0.5266–3.3264
Pelvis	0.65158	0.48411	1.8116	0.1783	1.9186	0.7428–4.9552
Extremities	0.69215	0.56456	1.5030	0.2202	1.9980	0.6607–6.0417
Skull	−1.05102	0.63274	2.7591	0.0967	0.3496	0.1011–1.2082
Radiologic pattern	0.47916	0.36753	1.6997	0.1923	1.6147	0.7857–3.3185
Stable or decreased ALP	2.36373	0.44600	28.0881	<0.0001	10.6305	4.4352–25.4800

ALP, Alkaline phosphatase.

## Data Availability

The data presented in this study are available on request from the corresponding author.
